# The effects of taurolidine alone and in combination with doxorubicin or carboplatin in canine osteosarcoma *in vitro*

**DOI:** 10.1186/1746-6148-9-15

**Published:** 2013-01-18

**Authors:** Kevin Marley, Stuart C Helfand, Wade A Edris, John E Mata, Alix I Gitelman, Jan Medlock, Bernard Séguin

**Affiliations:** 1 Department of Clinical Sciences, Oregon State University, Corvallis, USA; 2 Department of Biomedical Sciences, Oregon State University, Corvallis, USA; 3 Department of Statistics, Oregon State University, Corvallis, Oregon 97331, USA; 4 Present address: Department of Cellular and Molecular Physiology, Milton S Hershey Medical Center Penn State University, 500 University Dr. Hershey, Pennsylvania 17033, USA; 5 Department of Clinical Sciences, Colorado State University, Fort Collins, Colorado 80523, USA

**Keywords:** Taurolidine, Osteosarcoma, In vitro, Apoptosis, Doxorubicin, Carboplatin

## Abstract

**Background:**

Osteosarcoma (OS) affects over 8000 dogs/year in the United States. The disease usually arises in the appendicular skeleton and metastasizes to the lung. Dogs with localized appendicular disease benefit from limb amputation and chemotherapy but most die within 6–12 months despite these treatments. Taurolidine, a derivative of taurine, has anti-tumor and anti-angiogenic effects against a variety of cancers. The following *in vitro* studies tested taurolidine as a candidate for adjuvant therapy for canine OS. Tests for p53 protein status and caspase activity were used to elucidate mechanisms of taurolidine-induced cell death.

**Results:**

Taurolidine was cytotoxic to osteosarcoma cells and increased the toxicity of doxorubicin and carboplatin *in vitro*. Apoptosis was greatly induced in cells exposed to 125 μM taurolidine and less so in cells exposed to 250 μM taurolidine. Taurolidine cytotoxicity appeared caspase-dependent in one cell line; with apparent mutant p53 protein. This cell line was the most sensitive to single agent taurolidine treatment and had a taurolidine-dependent reduction in accumulated p53 protein suggesting taurolidine’s effects may depend on the functional status of p53 in canine OS.

**Conclusion:**

Taurolidine’s cytotoxic effect appears dependent on cell specific factors which may be explained, in part, by the functional status of p53. Taurolidine initiates apoptosis in canine OS cells and this occurs to a greater extent at lower concentrations. Mechanisms of cell death induced by higher concentrations were not elucidated here. Taurolidine combined with doxorubicin or carboplatin can increase the toxicity of these chemotherapy drugs and warrants further investigation in dogs with osteosarcoma.

## Background

Osteosarcoma (OS) is the most common cancer of bone in dogs [1]. Tumors usually arise in the appendicular skeleton and metastasize to the lung. In cases with localized appendicular disease, amputation of the affected limb along with chemotherapy can extend life expectancy up to about 12 months on average but there is no cure. An increased understanding of the pathogenesis of OS is needed in order to facilitate the development of more effective therapeutic strategies. One such strategy is to combine drugs with different mechanisms of action in an attempt to overcome tumor heterogeneity and drug resistance. In this regard, it is reasonable to consider adding new antineoplastic drugs to the treatments of patients receiving traditional chemotherapeutics and one such candidate for this is the antimicrobial and antineoplastic drug taurolidine [2,3].

Taurolidine is a derivative of the amino acid taurine that has anti-tumor and anti-angiogenic effects against a variety of cancers [4-6]. Originally developed for its antibacterial properties, taurolidine has few detrimental effects on normal cells [7] but inhibits cancer cell proliferation [8] and tumor growth by inducing apoptosis [5,9-12] possibly through p53-dependent mechanisms [13]. Taurolidine modulates apoptosis by altering Bcl-2/Bax concentrations [9], and exerts anti-metastatic effects within the tumor microenvironment through inhibition of angiogenesis and endothelial cell adhesion [14]. Taurolidine appears to induce autophagy and necroptosis in glioma cell lines [15] and has been used to treat glioblastoma and gastric carcinoma in humans [16,17]. This study tested taurolidine against human and canine osteosarcoma cell lines to determine its cytotoxicity and potential as an adjuvant therapy for doxorubicin or carboplatin treatment in dogs with OS.

## Methods

### In vitro testing

Four canine and one human OS cell lines were used in this study. They included canine D17 (ATCC CCL183), Clone 4, developed in our laboratory [18], COS [19], and HMPOS [20], and the human cell line SAOS-2 (ATCC HTB-85) which was included to establish consistency with reports using human cell lines. Cells were cultured at 37°C in a humidified 5% CO_2_ atmosphere in RPMI 1640 (Invitrogen) supplemented with 2mM glutamine, 2mM sodium pyruvate, 2mM HEPES, 1% pen-strep, and 10% fetal bovine serum. Taurolidine (2%) with and without 5% polyvinylpyrrolidone (PVP) was kindly provided by TauroPharm GmbH (Waldbüttelbrunn, Germany). Doxorubicin (2mg/ml) and carboplatin (10 mg/ml) were individually diluted 1:200 in RPMI 1640 prior to use. Combinations of taurolidine with doxorubicin or carboplatin were tested against SAOS-2, Clone 4, and D17 cell lines.

Cell viability was assessed using an MTS assay (Promega). Cells were seeded in 96-well plates at a density of 2500 or 5000 cells per well and allowed to adhere overnight. The medium was then replaced with fresh medium containing various concentrations of taurolidine, and/or doxorubicin or carboplatin. Absorbance was measured at λ490 after 60 minutes, 37°C, incubation with the MTS product. Data are expressed as means ± SD of triplicate wells, or quadruplicate wells, for the carboplatin experiments and are representative of at least two independent experiments.

The major effects of taurolidine were observed after 24 hrs incubation; therefore taurolidine-only experiments were performed using a 24 hr incubation period and 5000 cells/well in the 96-well plates. The doxorubicin and carboplatin experiments required 72 hr incubation periods so these were seeded with fewer cells (2500) to limit cell contact inhibition.

Our initial experiments used taurolidine that contained 5% PVP which is used as a stabilizing agent for storage purposes. Once we learned that dogs are allergic to PVP, we changed to taurolidine without PVP. Thus taurolidine without PVP was used for all subsequent experiments, which were those that included carboplatin.

Apoptotic changes in target cells were detected using three independent methods. These included a colorimetric ELISA based on the detection of nucleosomes formed as a result of DNA fragmentation, flow cytometric detection of early apoptotic changes in the cell membrane, and by Western blot for PARP cleavage, which is an indirect indication of caspase activity. For the nucleosome ELISAs, cells were initially treated as described above. After 12, and 24 hrs exposure to 125 μM, 250 μM taurolidine or PVP alone, culture supernatants were removed and processed using a commercially available kit (Roche). Absorbance was measured at λ405.

Flow cytometry was used to detect binding of annexin-V to phosphotidyl serine on the surface of cells in early apoptosis and propidium iodide to DNA in late stage apoptosis or dead cells. Tumor cells were incubated with 125 μM taurolidine and collected, using 0.005% trypsin in PBS to release the adherent cells, after 4, 24, and 48 hrs and reacted with biotinylated annexin V-FITC and propidium iodide using a commercially available kit (Calbiochem). A minimum of 20,000 cells per sample were collected on a Beckman Coulter FC-500 flow cytometer. Data analysis and software compensation were performed using WinList (Verity Software, Topsham, ME).

PARP cleavage, a surrogate marker of caspase activity, was determined by Western blot using cells that had been exposed to two concentrations of taurolidine with and without prior incubation with the general caspase inhibitor Z-VAD-FMK (CalBiochem ), as described [10]. Briefly, cells were seeded in 6-well plates (3 × 10^6^/well) and allowed to adhere overnight. Cells were incubated in 25 μM Z-VAD-FMK or DMSO in supplemented RPMI medium for 60 min. The medium was changed and the cells were subsequently incubated in 0, 125 or 250 μM taurolidine in supplemented RPMI for 12 hrs and processed as described below.

Cells were seeded (3 × 10^6^/well) in 6-well plates and allowed to adhere overnight prior to incubation as described above and in figure legends. At the end of the incubation period, the media and cells were removed, using a cell scraper, and the cells were pelleted in a tabletop centrifuge (3 min, 1200 × g). Cell pellets were rinsed by re-suspending twice in 3 mls ice cold PBS and extracted in 50 μl ice cold RIPA buffer with protease inhibitor cocktail (Sigma). Extracts were sonicated four times (1 sec each) using an ultrasonic dismembranator (Fisher, Model 150T) and pelleted at 10,000 × g to remove cellular debris. Protein concentration was measured using a Bradford assay (Bio-Rad). Proteins (20 μg/lane) were separated on 4-12% SDS polyacrylamide gels and transferred to PVDF membranes using standard methods. The membranes were blocked in 1.5% albumin and reacted with indicated primary antibodies diluted 1:200 for 3 hrs at room temperature (antibodies from Santa Cruz Biotechnology; PARP, sc-13628; p53, \sc-136023; actin, sc-47778). The membranes were washed, reacted with horseradish peroxidase-linked secondary antibody (Santa Cruz Biotechnology, sc-2005) diluted 20,000:1, and exposed to substrate (Thermo Scientific).

Because D17 cells expressed minimal amount of PARP, we performed an immunoprecipitation (IP) prior to Western blots as described above. Briefly, 100 μg PARP antibody was suspended in 1 ml of IP buffer (25 mM Tris, 150 mM NaCl; pH 7.2) and reacted overnight with protein A/G linked agarose beads (Thermo Scientific). The following day, 1 mg of protein from whole cell extracts was added and allowed to react at room temperature for 2 hours. The beads were rinsed several times in IP buffer and immune complexes were eluted in 50 ul SDS PAGE loading buffer and separated as described above for Western blots.

Nuclear accumulation of p53 protein is an indication of mutation in the p53 gene [21,22]. As an initial screen for possible p53 mutants, we used immunocytochemistry to detect abnormal expression of p53 in four canine OS cell lines. Normal canine lymphocytes obtained from a fine needle aspirate of a lymph node from a healthy dog were used as a control. Cells were pelleted onto positively charged slides and allowed to air dry. The slides were subsequently fixed in acetone, air dried and stained according to standard methods [23] using a monoclonal antibody against p53 protein (sc-136023), chromagen Nova Red (Vector Laboratories) as a secondary and hematoxylin (Dako S3309) as the counter stain. Stained cells were imaged on a microscope and scoring was performed analogous to the Hercep Test system [24]. Briefly, two independent individuals scored the staining intensity of each slide in several representative fields using a subjective scoring criteria consisting of a rating of 0 for no staining to a rating of 3 for strong positive staining in all cells.

Phase contrast photomicrographs were obtained with an inverted microscope (Eclipse ti, Nikon, Melville, NY) using the 40X objective lens, and a digital camera. Images were processed using NIS-Elements software (Nikon, Melville, NY).

### Data analyses

**Figure 1:**
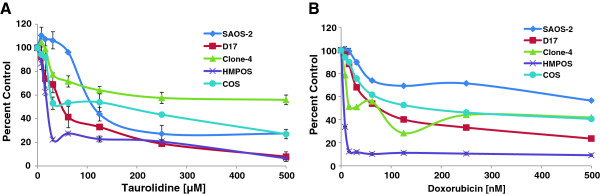
**Effects of taurolidine or doxorubicin on proliferation of OS cell lines.** Cells were incubated for 24 hrs with taurolidine **A**) or 72 hrs with doxorubicin **B**) and cytotoxic effects were determined using an MTS assay. Error bars indicate SD of 4 replicate wells. In some cases error bars are too small to be visible.

**Figure 2:**
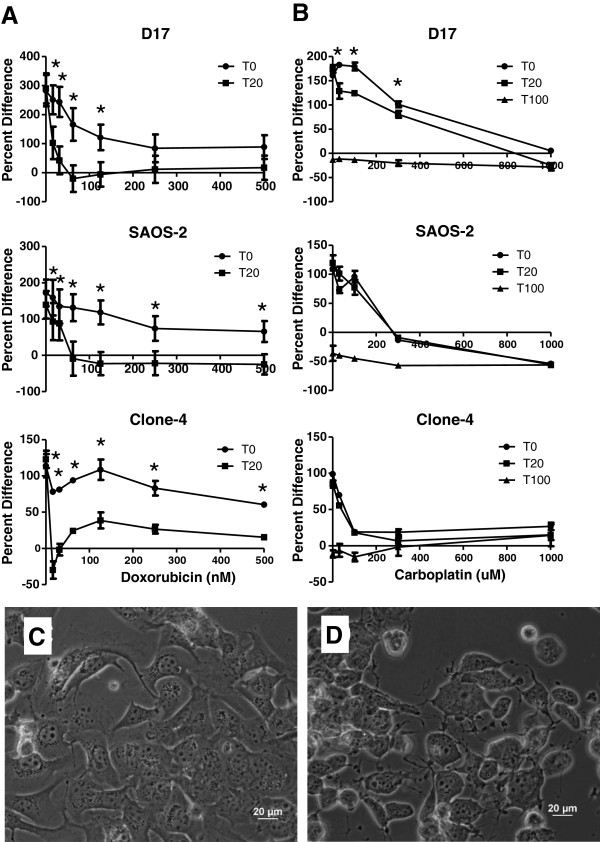
**Effects of doxorubicin or carboplatin ± taurolidine on proliferation of OS cell lines.** Three OS cell lines were incubated for 72 hrs in increasing concentrations of doxorubicin **A**) or carboplatin **B**) in the presence or absence of taurolidine and cytotoxic effects were evaluated using an MTS assay. T0 indicates no taurolidine while T20 and T100 indicate 20 μM and 100 μM taurolidine respectively. Error bars indicate SD of 4 replicate wells. In some cases error bars are too small to be visible. * indicates a synergistic interaction (see methods). Data are presented as percent difference from time 0 on the y-axis (see methods and results). Photomicrographs of D17 cells after 6 hr incubation with 100 nM doxorubicin **C**) or with 100 nM doxorubicin plus 20 μM taurolidine. **D**) Cytoplasmic contraction is readily appreciated in the cells exposed to the drug combination and represents an early apoptotic effect.

The 50% inhibitory concentrations (IC50s) were calculated with Prism Graphpad software using non-linear regression and the log of the inhibitor versus variable slope response equation. Constraints were set at 100% for the top and 0 and −0.5 for the baselines in Figures 1 and 2 respectively.

The type of interaction between taurolidine and doxorubicin or carboplatin was determined using the following equations [25]:

$$\text{Synergistic}={\text{SF}}_{t+y}<{\text{SF}}_{t}{\text{xSF}}_{y}$$

$$\text{Additive}={\text{SF}}_{t+y}={\text{SF}}_{t}{\text{xSF}}_{y}$$

$$\text{Sub}-\text{additive}={\text{SF}}_{t}{x\;\text{SF}}_{y}<{\text{SF}}_{t+y}<{\text{SF}}_{t}{\text{andSF}}_{y}$$

$$\text{Antagonistic}={\text{SF}}_{\text{t}+y}>{\text{SF}}_{t}{\text{orSF}}_{y}$$

SF_t+y_ = surviving fraction of cells exposed to the combination of taurolidine and doxorubicin or carboplatin, SF_t_ = surviving fraction of cells exposed to taurolidine alone, SF_y_ = surviving fraction of cells exposed to doxorubicin or carboplatin alone. Interactions were determined only for those combinations which led to a surviving fraction of cells that were significantly different than those of each individual drug at the same respective concentration. These equations are appropriate provided the effect is anti-proliferative [26], a condition that was met when the interaction was determined except in cell line D17. At low concentrations (30 and 100 μM), carboplatin had pro-proliferative effects in cell line D17. For these concentrations, the SF of the control group was used in the equation for the SF of carboplatin as it was the most stringent conditions to evaluate the drug interaction.

Univariate ANOVA with Tukey’s posthoc tests, when appropriate, were performed using PASW-17 software. Data in Figure 2A and 2B are normalized to a baseline absorbance value taken at the initiation of the drug incubation period, to account for the cell count at time zero, and expressed as percent difference on the y-axis.

**Figure 3:**
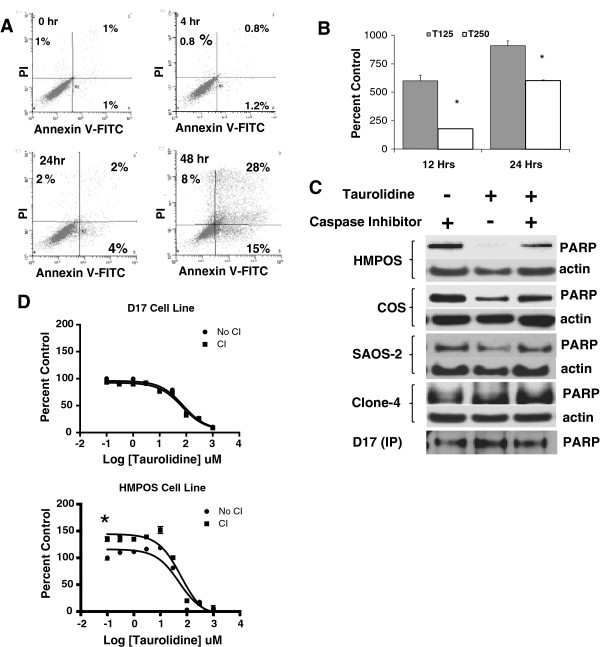
**Mechanisms of** t**aurolidine-induced cytotoxicity are cell line and concentration dependent.** Binding of annexin-V-biotin-FITC and propidium iodide (PI) to taurolidine-treated D17 OS cells was determined by 2-color flow cytometry. **A**) Data are displayed as dot plots and data points in the lower right quadrant (i.e., annexin-V positive) represent early apoptotic cells while data points in the upper left quadrant represent necrotic cells and points in the upper right quadrant (dual annexin-V/PI positive) represent cells in late apoptosis and necrosis. **B**) The release of nucleosomes as an indicator of apoptosis following taurolidine treatment of D17 OS cells was determined at two time points. Data are expressed as percent of apoptosis values measured in untreated cells at the same time points. The higher taurolidine concentration (250 μM) was less effective at inducing apoptotic changes in this assay indicating cell death occurred by other mechanisms. T125, T250 = 125 μM and 250 μM taurolidine respectively. * indicates different from 125 μM at same time point (p < .01). Error bars indicate SD. **C**) Western blots show degradation of PARP as an indirect indicator of taurolidine-activated caspase activity in COS and HMPOS cells. Cells incubated 12 hrs in taurolidine as described in methods. No loading control was available for the D17 blot because it was an immunoprecipitation. **D**) These proliferation assays show the caspase inhibitor (CI) Z-VAD-FMK protects HMPOS but not D17 OSA cells from caspase-dependent cell death at low concentrations of taurolidine, consistent with PARP cleavage seen in 3C. * indicates intercept is different between treated and untreated conditions (P < .05).

Flow cytometry data (Figure 3A) were analyzed using Chi-squared tests to compare differences in necrosis versus apoptosis. Data shown in Figure 3D were analyzed using linear regression with Prism GraphPad, software (La Jolla, Ca). P values ≤ .05 were considered statistically significant.

## Results

### Taurolidine-induced cytoxicity

The potential effects of PVP alone were assessed by comparing untreated cells with cells treated with PVP at the same concentration as those in the high taurolidine condition. This amounted to 0.07% PVP which had no discernible effect on the cell lines or the MTS assay (data not shown). Taurolidine had concentration-dependent cytotoxic effects against all cell lines tested (Figure 1A). The intensity of the cytotoxic effects of doxorubicin were also cell line dependent with the major effects of the drug occurring at the lower concentrations and little additional benefit from higher concentrations of drug (Figure 1B). The IC50s for taurolidine and doxorubicin alone are shown in Table 1. Figure 2 shows the cytotoxic effects of doxorubicin or carboplatin alone and in combination with taurolidine. Data in Figure 2A and B are graphed using percent difference along the y-axis. Thus, cells that have doubled in number have a y value of 100. Zero indicates no change in cell number and values below 0 indicate cell numbers were lower than they were at the beginning of the experiment. Graphed in this way, it can be seen that 20 μM taurolidine enhanced the effect of doxorubicin but the best that could be expected under these conditions would be, on average, zero growth. Adding 20 μM taurolidine to carboplatin enhanced the effect of carboplatin alone but only in the D17 cell line. In most cases, the 100 μM taurolidine plus carboplatin combinations resulted in fewer remaining cells than were present at time zero (y-axis values < 0). The IC50s for the drug combination studies are shown in Table 2. The photomicrographs in Figure 2C and D show doxorubicin and doxorubicin plus taurolidine treated D17 cells (respectively) after 6 hours drug incubation. Only the doxorubicin-taurolidine combination induced cytoplasmic contraction which is an early indicator of apoptosis [23].

**Table 1:** **IC50s for human and canine osteosarcoma cell lines treated with taurolidine or doxorubicin (inhibition curves shown in Figure**1**)**

**Cell line**	**Taurolidine (μM)**	**Doxorubicin (nM)**
Saos-2	150	NR
D17	55	92
Clone-4	NR	61
HMPOS	23	1
COS	119	119

NR = IC50 Not Reached.

**Table 2:** **IC50s for human and canine osteosarcoma cell lines treated with doxorubicin or carboplatin ± taurolidine (inhibition curves shown in Figure**2**A and**2**B)**

**Cell line**	**Doxorubicin (nM)**	**Doxorubicin (nM) + 20 μM Taurolidine**	**Carboplatin (μM)**	**Carboplatin (μM) + 20 μM Taurolidine**	**Carboplatin (μM) + 100 μM Taurolidine**
Saos-2	252	22	198	204	0
D17	100	12	414	202	0
Clone-4	NR	4	538	134	0

NR = IC50 Not Reached.

### Taurolidine-induced apoptosis

Annexin V-FITC and propidium iodide flow cytometry was used to quantify the proportion of D17 cells undergoing apoptosis and necrosis in response to 125 μM taurolidine at three time points (Figure 3A). Apoptotic changes predominated over necrosis during the early stages of taurolidine cytotoxicity (p < .01). After 48 hours, 15% of D17 cells demonstrated early apoptotic changes and 28% had progressed to late apoptotic or necrotic stages (Figure 3A). The release of nucleosomes from D17 cells was also used to evaluate the apoptotic response to 125 μM and 250 μM taurolidine (Figure 3B). The lower concentration of taurolidine triggered a greater apoptotic response at both the 12 and 24 hour time points (p < .05). The 125 μM concentration was also more effective at inducing caspase activity as indicated by PARP cleavage (Figure 3C). The HMPOS cells showed the greatest response with complete PARP cleavage when exposed to 125 μM taurolidine. PARP cleavage was not observed in HMPOS cells exposed to 250 μM taurolidine (data not shown). The addition of caspase inhibitor (Z-VAD-FMK) blocked PARP cleavage under these conditions indicating that 125 μM taurolidine induces apoptosis by activating the mitochondrial cytochrome-c caspase cascade [27]. This response was present but considerably less pronounced in COS and SAOS-2 cells and was not seen in the D17 and Clone 4 cell lines. Because taurolidine induced PARP cleavage in the HMPOS but not D17 cells, we investigated taurolidine-induced cytotoxicity with and without caspase inhibition using an MTS cytotoxicity assay. Figure 3D shows the caspase inhibitor had no effect on the cytotoxic effect of taurolidine on D17 cells but protected HMPOS cells from death in the presence of low concentrations of taurolidine. These results indicate that taurolidine exerts cytotoxic effects through multiple mechanisms that are both caspase-dependent and independent. We hypothesized these differences might be modulated by the mutational/functional status of p53.

### Taurolidine effects on expression of p53 protein in OS cell lines

**Figure 4:**
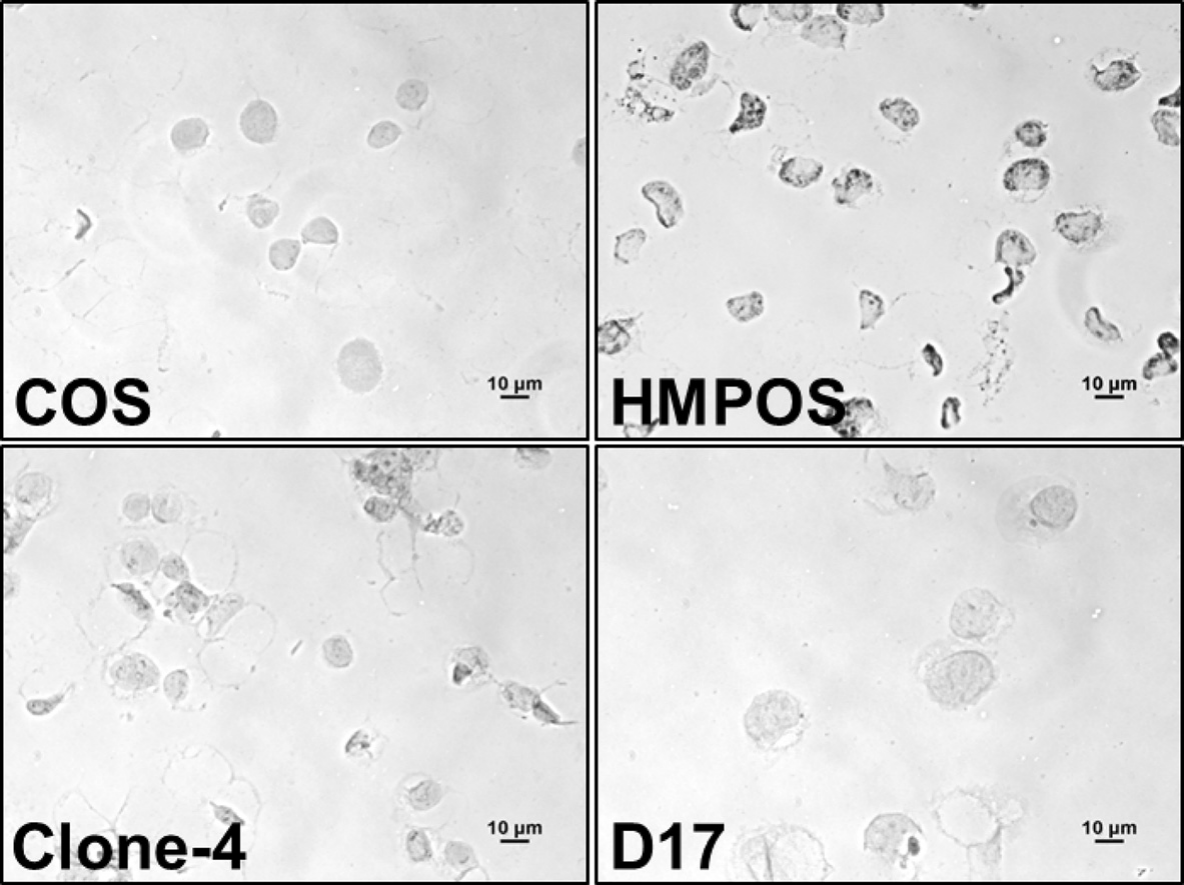
**p53 protein accumulation in untreated osteosarcoma cells.** Representative photomicrographs of cells probed with an antibody against p53 protein show strong accumulation in HMPOS cells, moderate staining in Clone-4 and none in the D17 and COS cell lines. The signal that can be observed in D17 and COS cells is the counterstain hematoxylin (see methods).

**Figure 5:**
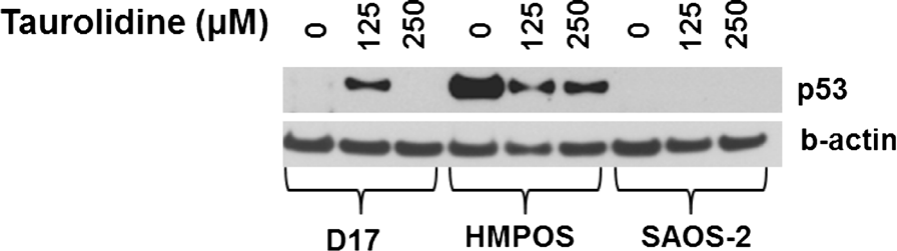
**Effects of taurolidine on p53 protein expression.** Western blots showing the effects of taurolidine exposure on this cell cycle regulatory protein is concentration and cell line dependent. The taurolidine-induced appearance of p53 protein in D17 cells suggests these cells proceed through a p53-induced apoptotic pathway when exposed to 125 uM taurolidine. In HMPOS cells, with apparent mutant p53, p53 was down-regulated suggesting cell death may occur through different pathways in this cell line. Clone 4 and COS cells did not show any p53 expression at any concentration of taurolidine tested (data not shown).

Because mutant p53 is responsible for evasion of apoptosis in many cancers [28], we sought to determine the role of p53-triggered apoptosis in response to taurolidine treatment. First, immunocytochemistry was used to evaluate canine OS cell lines for p53 expression in resting cells. Data shown in Figure 4, and summarized in Table 3, suggest p53 protein accumulates strongly in HMPOS, mildly in Clone-4 and is absent in COS and D17 cells. Taurolidine treatment caused a dramatic decrease in p53 protein expression in HMPOS but an opposite effect in D17 cells where protein expression strongly increased (Figure 5). We interpret this to indicate the possibility that p53-mediated apoptosis in response to taurolidine proceeds appropriately in D17, but not in HMPOS cells [22,28].

**Table 3:** Immunocytochemistry scores for p53 protein accumulation. 0 indicates no detection and +++ indicates strong staining in all cells

**Cell line**	**Score**
D17	0
Clone 4	++
HMPOS	+++
COS	0

## Discussion

Osteosarcoma continues to be a major cause of death for dogs worldwide. Often, microscopic metastases exist at diagnosis and these lead to a dismal prognosis. Early treatment with chemotherapy and surgery prolongs survival but unacceptable drug toxicity prevents dose escalation to levels capable of reversing metastatic disease. New drug treatments are therefore desperately needed. In the present studies we investigated taurolidine alone, and in combination with doxorubicin or carboplatin *in vitro* as a potential adjuvant therapy for canine OS. The IC50s reported here are similar to those reported for human OS cell lines [10] and a maximal apoptotic response was observed at a concentration of 125 μM, which is a clinically achievable concentration in both humans [29] and dogs (Seguin unpublished results). Drug levels above 125 μM had little, if any, additional benefit and cell death at these concentrations appeared to occur by a mechanism other than apoptosis. This is consistent with previously reported findings that showed taurolidine promotes apoptosis at lower concentrations and necrosis at higher concentrations [11].

The most commonly used chemotherapeutic agents for OS in dogs are doxorubicin, carboplatin, and cisplatin [30] although doxorubicin and carboplatin are currently preferred because cisplatin is highly nephrotoxic in dogs. Accordingly we sought to determine the potential for a synergistic interaction when exposing cells to taurolidine and doxorubicin or carboplatin. Only certain combinations of taurolidine and doxorubicin or taurolidine and carboplatin achieved a surviving fraction (SF) of cells that was different from that of each drug by itself (Figure 2A and B). When the SFs were different, the interaction between the drugs was synergistic. In the experiments with carboplatin we chose to also test taurolidine at 100 μM to reflect the concentration achieved in the serum of dogs (Seguin, unpublished data). At that concentration, taurolidine alone was so effective that in essence adding carboplatin could not improve on those results (Figure 2B). Our drug combination studies demonstrate taurolidine can be combined to enhance the sensitivity of OS cells to doxorubicin or carboplatin *in vitro.* Optimal drug concentrations and incubation periods appeared cell line dependent and were not fully elucidated here.

Once we learned that dogs are allergic to PVP, we changed to taurolidine without PVP to better reflect the potential clinical use of taurolidine in dogs. PVP is a stabilizing agent for storage purposes. Our experiments indicated that PVP alone did not have cytotoxic activity against OS cells in vitro. It is possible that without PVP, the taurolidine solution could lose its biologic activity. However our results show that taurolidine retains its cytotoxic activity in the absence of PVP.

We performed two independent assays of apoptosis that show the cytotoxic effects of taurolidine at 125 μM proceeds through apoptotic mechanisms in canine OS cells. Our results reveal that apoptosis begins within 4 hours of taurolidine exposure and that response to taurolidine depends on the cell line being tested. We speculated the cell line-specific differences could be, in part, due to p53 functional status. Our results support this hypothesis although definitive studies remain to be carried out in this regard.

## Conclusion

Taurolidine is cytotoxic to canine OS *in vitro* and has the potential to enhance the cytotoxicity of doxorubicin or carboplatin in animals with OS. Preliminary clinical trials to test this hypothesis are currently underway in our facility.

## Abbreviations

OS: Osteosarcoma; PVP: Polyvinylpyrrolidone; PARP: Poly (ADP-ribose) polymerase; DMSO: Dimethyl sulfoxide; RIPA: Radio-Immunoprecipitation Assay; PVDF: Polyvinylidene fluoride; SF: Surviving fraction.

## Competing interests

The authors declare that they have no competing interests.

## Authors’ contributions

KM carried out the experiments, analyzed and interpreted the data, and drafted the manuscript. SCH helped design the experiments, with interpretation of data and revision of manuscript for intellectual content. WAE carried experiments, analyzed and interpreted data. JEM helped with experiment design, data collection and analysis. AIG helped with the statistical analysis of the data. JM helped with data analysis. BS was responsible for conception of study, design of experiments, interpretation of data and revision of manuscript for intellectual content. All authors read and approved the final manuscript.
